# Satellite Constellation Optimization for Emitter Geolocalization Missions Based on Angle of Arrival Techniques

**DOI:** 10.3390/s25113376

**Published:** 2025-05-27

**Authors:** Marcello Asciolla, Rodrigo Blázquez-García, Angela Cratere, Vittorio M. N. Passaro, Francesco Dell’Olio

**Affiliations:** 1 Department of Electrical and Information Engineering, Politecnico di Bari, Via Orabona 4, 70126 Bari, Italy; a.cratere@phd.poliba.it (A.C.); vittorio.passaro@poliba.it (V.M.N.P.); 2 Information Processing and Telecommunications Center (IPTC), Universidad Politécnica de Madrid, Av. Complutense 30, 28040 Madrid, Spain; rodrigo.blazquez@upm.es

**Keywords:** angle of arrival, dilution of precision, geolocation, low earth orbit, mission, optimization, satellite, simulation, space sensors, space systems

## Abstract

The context of this study is the geolocation of signal emitters on the Earth’s surface through satellite platforms able to perform Angle of Arrival (AOA) measurements. This paper provides the theoretical framework to solve the optimization problem for the orbital deployment of the satellites minimizing the variance on the position error estimation with constraints on the line of sight (LOS). The problem is theoretically formulated for an arbitrary number of satellites in Low Earth Orbit (LEO) and target pointing attitude, focusing on minimizing the Position Dilution of Precision (PDOP) metric, providing a methodology for translating mission design requirements into problem formulation. An exemplary numerical application is presented for the operative case of the placement of a second satellite after a first one is launched. Simulation results are on angles of true anomaly, right ascension of the ascending node, and spacing angle, while accounting for orbital radius and emitter latitude. New insights on trends, parameter dependencies, and properties of symmetry and anti-symmetry are presented. The topic is of interest for new technological demonstrators based on CubeSats with AOA payload. Civil applications of interest are on interceptions of non-cooperative signals in activities of spectrum monitoring or search and rescue.

## 1. Introduction

The scenario of this study considers two actors: an emitter of electromagnetic waves located on the Earth’s surface and a set of satellite platforms with receivers acting as sensors to geolocate the emitter.

Geolocation is the process of determining the physical location of an entity in geographical coordinates. This process is crucial for many applications, ranging from navigation and communication to surveillance and environmental monitoring [1].

In general, among the cases of interest in which it is important to perform an operation of geolocation, there is the one where the signal is non-cooperative; a signal is said to be non-cooperative when its position (and the signal itself) are not supposed to be known. It can be intentional—for example, it can be the case of illegal activities such as the jamming of signals used by civil aviation—or it can be non-intentional, such as the case of emergency calls from remote locations. In the first case, the interception and geolocation of the signal is used in patrolling activities of spectrum monitoring, while, in the latter case, it is used to coordinate operations of search and rescue to help people in need [2].

This study considers satellite systems with passive payloads, comprising only receivers able to capture electromagnetic waves without the use of onboard transmitters or radars [3,4].

In general, this kind of geolocation can be accomplished through various methods, one of which is based on the estimation of the Angle of Arrival (AOA) [1,2,3,5], i.e., the angle at which a signal arrives at a receiver. One of the most common techniques for AOA estimation is array beamforming [4].

Other classical sensing techniques are Time Difference of Arrival (TDOA), Frequency Difference of Arrival (FDOA), and Received Signal Strength (RSS) [1,2,3,5].

For antenna array technology, recent trends and interests are on innovative analysis and synthesis considering the minimization of the number of elements [6], beam pattern design and rotating elements [7], error calibration for space applications [8], quantum antenna array [9], and machine learning applied to AOA estimation and processing [10].

By combining AOA measurements from multiple spatially distributed receivers, it is possible to triangulate the precise location of the signal emitter. This method is based on the principle that signals arrive at different angles to different receivers depending on the emitter’s location. In general, in order to perform the geolocation of the signal, at least two separate measurement directions are needed.

In satellite-based geolocation systems, the measurement is primarily affected by two sources of error.

The first source is associated with the individual signal receivers and includes contributions from time delays [11], relativistic effects [12], the presence of the atmosphere [13], satellite attitude, antenna technology [4], and the signal-to-noise ratio (SNR) [5,14]. Among these, SNR and the electronic performance of the receiver are the most significant contributors and are often modeled using the Cramér-Rao Lower Bound (CRLB) [15].

The second source of error has a geometric origin and it is known as Dilution of Precision (DOP), which arises from the spatial distribution of receivers in space. The minimization of the DOP enhances the overall precision of the geolocation system and improves the services that rely on satellite-based geolocation. The optimization of the spatial distribution of satellites in constellations started in the context of Global Navigation Satellite Systems (GNSSs) [16], although for these kinds of systems, the measurement is based on Time of Arrival.

In addition to GNSS studies, some works have focused on terrestrial wireless systems exploring fixed geometries in a plane (e.g., triangular, squared, and hexagonal distributions [17]) as well as optimal geometrical distributions of receivers in two dimensions [18,19]. Other studies have derived closed-form solutions for estimation errors [20], developed closed-form estimators for hybrid measurements (i.e., combining AOA with other techniques), and explored the simultaneous localization of multiple emitters [21].

Recent mathematical analyses, driven by the availability of different GNSS systems, have focused on determining how many satellites minimize the DOP for GNSS systems [22,23], reducing computational costs [24], and developing approximate solutions using artificial intelligence in non-line-of-sight conditions [25].

The primary aim of this paper is to present a mathematical optimization model for the 3D spatial distribution of clusters of satellites in LEO, where the line of sight (LOS) constitutes a strong constraint. Previous investigations by the authors on this topic were presented in [26], where properties of symmetry and anti-symmetry related to the right ascension of the ascending node, the true anomaly, and the spacing angle of optimal deployments were predicted in simplified Earth-scanning mission scenarios.

In this study, a theoretical model of constrained optimization was derived to account for an arbitrary number of satellites in LEO, capable of performing AOA measurements in case of target pointing and considering an emitter of signal on the surface of the Earth.

An alternative formulation of the problem in terms of differences is introduced, simulated, and analyzed for a two-satellite configuration, incorporating variations in orbit radius and emitter latitude.

The motivation behind this study is related to a set of operative problems on the update of an existing constellation. In this context, the developed framework considers the operative example, which consists of the optimal placement of a second companion satellite after the first one is launched.

These activities are of interest for a set of new technological demonstrators based on CubeSats platforms and the miniaturization of arrays of antennas. One example of these missions is the SAILS (Spaceborne Autonomous Identification and Localization System) project supervised and funded by the Italian Space Agency, in which the Polytechnic University of Bari is collaborating as a scientific partner [27].

Section 2 describes the mathematical model of the spatial representation of the satellites and the signal emitter, along with the measurement and orbital models, and outlines the general optimization problem. Section 3 presents the optimization problem for two satellites, its alternative formulation in terms of differences, the main results of the simulations, and their analysis. Section 4 includes the conclusions of this study. Appendix A presents a glossary for the mathematical notation used in this paper.

## 2. Mathematical Modeling

### 2.1. Spatial Representation

To solve the mathematical problem, the spatial representation of the objects of interest is needed. The signal emitter is denoted by *E* and the satellites are denoted by $S_{1},S_{2},\ldots ,S_{k},\ldots ,S_{n}$, where *n* is the total number of satellites. Both the signal emitter and the receiving satellites are represented as geometrical points without spatial extension.

In general, when the signal emitter is located on Earth, it is typically represented by geodetic latitude Λ, longitude λ, and height *h* (LLH) as a set of coordinates: ${\left.x_{E}\right|}_{\mathrm{LLH}}={\left(\Lambda ,\lambda ,h\right)}^{T}$. It can be recalled that geodetic latitude is defined as the angle between the equatorial plane and the normal unit vector, which is normal at the considered point on the representative ellipsoid of Earth’s surface.

Instead, the *k*-th satellite is generally expressed in perifocal coordinate system (PQW) using orbital parameters, as shown in (1), where $a_{k}$ is the semi-major axis, $e_{k}$ is the orbit eccentricity, and $\nu_{k}$ is the true anomaly [28,29]. The perifocal coordinate system is a coordinate system used to represent the orbit of a satellite. It has its center in the Earth, which is in one of the foci of the elliptic orbit of the satellite, and is denoted by the PQW acronym because the unit vectors are commonly denoted by the letters p, q, and w; in particular, p is directed towards the periapsis of the orbit, q belongs to the plane of the orbit and is spaced by 90° from p, while the third one is obtained from the cross product w=p×q and is orthogonal to the plane of the orbit.

(1) $${\left.x_{S_{k}}\right|}_{\mathrm{PQW}}=\frac{a_{k}\left(1-e_{k}^{2}\right)}{1+e_{k}\cos\left(\nu_{k}\right)}\left(\begin{array}{c} \cos\left(\nu_{k}\right)\\ \sin\left(\nu_{k}\right)\\ 0 \end{array}\right)\;k=1,2,\ldots ,n$$

For a satellite $S_{k}$, the data conversion from PQW to ECI occurs through the matrices of rotation $R_{\left(\Omega_{k},3\right)}$, $R_{\left(i_{k},1\right)}$, and $R_{\left(\omega_{k},3\right)}$, represented in (2). In particular, for the *k*-th satellite, $i_{k}$ is the orbit inclination, $\Omega_{k}$ is the right ascension of the ascending node, and $\omega_{k}$ is the argument of perigee; the second argument of the matrices is related to the axis of rotation.

(2) $$\begin{array}{c} R_{\left(\omega_{k},3\right)}=\left[\begin{array}{ccc} \cos\left(\omega_{k}\right) & -\sin\left(\omega_{k}\right) & 0\\ \sin\left(\omega_{k}\right) & \cos\left(\omega_{k}\right) & 0\\ 0 & 0 & 1 \end{array}\right]\;k=1,2,\ldots ,n\\ R_{\left(i_{k},1\right)}=\left[\begin{array}{ccc} 1 & 0 & 0\\ 0 & \cos\left(i_{k}\right) & -\sin\left(i_{k}\right)\\ 0 & \sin\left(i_{k}\right) & \cos\left(i_{k}\right) \end{array}\right]\;k=1,2,\ldots ,n\\ R_{\left(\Omega_{k},3\right)}=\left[\begin{array}{ccc} \cos\left(\Omega_{k}\right) & -\sin\left(\Omega_{k}\right) & 0\\ \sin\left(\Omega_{k}\right) & \cos\left(\Omega_{k}\right) & 0\\ 0 & 0 & 1 \end{array}\right]\;k=1,2,\ldots ,n \end{array}$$

PQW-to-ECI conversion is then reported as in (3).

(3) $${\left.x_{S}\right|}_{\mathrm{ECI}}=R_{\left(\Omega_{k},3\right)}R_{\left(i_{k},1\right)}R_{\left(\omega_{k},3\right)}{\left.x_{S}\right|}_{\mathrm{PQW}}\;k=1,2,\ldots ,n$$

For the emitter, data conversion follows two steps: from LLH to ECEF (Earth-Centered Earth-Fixed) and from ECEF to ECI.

Denoting with $e_{⊕}$ the eccentricity of Earth’s ellipsoid and with $a_{⊕}$ the ellipsoidal equatorial radius, the LLH-to-ECEF conversion [30] is reported as in (4). The value of the function $N\left(\Lambda \right)$ is reported in (5).

(4) $$x_{E}{|}_{\mathrm{ECEF}}=\left(\begin{array}{c} \left(N\left(\Lambda \right)+h\right)\cos\left(\Lambda \right)\cos\left(\lambda \right)\\ \left(N\left(\Lambda \right)+h\right)\cos\left(\Lambda \right)\sin\left(\lambda \right)\\ \left(N\left(\Lambda \right)\left(1-e_{⊕}^{2}\right)+h\right)\sin\left(\Lambda \right) \end{array}\right)$$

(5) $$N\left(\Lambda \right)=\frac{a_{⊕}}{1-e_{⊕}^{2}\sin\left(\Lambda \right)}$$

The most common ellipsoidal Earth model is the WGS84 model [31], but, in this study, a spherical model with a radius of $r_{⊕}=6371.000$ km is considered, so $e_{⊕}=0$, $a_{⊕}=N\left(\Lambda \right)=r_{⊕}$, and (4) becomes as represented in (6).

(6) $$x_{E}{|}_{\mathrm{ECEF}}=\left(\begin{array}{c} \left(r_{⊕}+h\right)\cos\left(\Lambda \right)\cos\left(\lambda \right)\\ \left(r_{⊕}+h\right)\cos\left(\Lambda \right)\sin\left(\lambda \right)\\ \left(r_{⊕}+h\right)\sin\left(\Lambda \right) \end{array}\right)$$

The conversion from ECEF to ECI is reported in (7), where $\alpha_{\mathrm{GR}}$ is the position of Greenwich meridian and it is a function of time *t*.

(7) $${x|}_{\mathrm{ECI}}=R_{\left(\alpha_{\mathrm{GR}}\left(t\right),3\right)}{x|}_{\mathrm{ECEF}}$$

Both the signal emitter and the satellites have to be expressed in the same reference system to write the optimization problem. The particular coordinate system for the calculations did not matter for the purposes of this study, and ECI was chosen simplicity because it is assumed to be inertial for the time span of a satellite mission and because both the transformations LLH-to-ECI for the signal emitter and PQW-to-ECI for the satellites are well known in the literature. It is noted for the reader that transformation between coordinate systems introduces computational errors: it could be possible to determine a new coordinate system that minimizes these computational errors, but this task is beyond the purposes of this study and, to the knowledge of the authors, it is an open problem that is never addressed for these kinds of applications.

In this study, the influence of dynamics in terms of forces/torques is absent, i.e., it is supposed that they are balanced and that the control system compensates for all the disturbances, and residual values are expected to have a secondary order effect.

### 2.2. Measurement Model

From a mathematical point of view, an AOA measurement of the emitter *E* by the *k*-th satellite $S_{k}$ is represented by the line of bearing (LOB), which can be expressed as $v_{\mathrm{LOB},k}=\left(x_{E}-x_{S_{k}}\right)/∥x_{E}-x_{S_{k}}∥$.

The solution to the geolocation problem is the unknown position of the emitter *E*, which is the junction of the lines of bearing. Generally speaking, at least two non-parallel lines of bearing are required for AOA measurements in contrast to other techniques. The least squares approach can be used to estimate the position when there is an error in the measurements.

The representation of the LOB in a local spherical coordinate system considers a set of two angles [32,33,34]. In this study, the two angles used to represent the measurement equation for the *k*-th satellite $S_{k}$ are the Azimuth angle (in the following with subscript Az) and Elevation angle (in the following with subscript El), denoted by $\alpha_{\mathrm{Az},k}$ and $\alpha_{\mathrm{El},k}$ respectively.

The representation of the LOB of the *k*-th satellite in a local spherical coordinate system is shown by (8).

(8) $$\left\{\begin{array}{c} x_{E}-x_{S_{k}}=\cos\left(\alpha_{\mathrm{Az},k}\right)\sin\left(\alpha_{\mathrm{El},k}\right)\;k=1,2,\ldots ,n\\ y_{E}-y_{S_{k}}=\sin\left(\alpha_{\mathrm{Az},k}\right)\sin\left(\alpha_{\mathrm{El},k}\right)\;k=1,2,\ldots ,n\\ z_{E}-z_{S_{k}}=\cos\left(\alpha_{\mathrm{El},k}\right)\;k=1,2,\ldots ,n \end{array}\right.$$

From (8), the angles of Azimuth $\alpha_{\mathrm{Az},k}$ and Elevation $\alpha_{\mathrm{El},k}$ can be obtained, as shown in (9).

(9) $$\left\{\begin{array}{c} \alpha_{\mathrm{Az},k}=\arctan\left(\frac{y_{E}-y_{S_{k}}}{x_{E}-x_{S_{k}}}\right)\;k=1,2,\ldots ,n\\ \alpha_{\mathrm{El},k}=\arctan\left(\frac{\sqrt{{\left(x_{E}-x_{S_{k}}\right)}^{2}+{\left(y_{E}-y_{S_{k}}\right)}^{2}}}{z_{E}-z_{S_{k}}}\right)\;k=1,2,\ldots ,n \end{array}\right.$$

For an array of antennas, the phase differences of the impinging electromagnetic wave in each array element are exploited to produce the measurement, which requires at least two elements per orthogonal direction. In particular, in this study, a cross array of antennas is considered.

An AOA measurement has many sources of error. From an engineering point of view, for a linear array of antennas on a satellite $S_{k}$, the most common evaluation is performed through the CRLB [15], which defines the minimum achievable standard deviations $\sigma_{{\hat{\varepsilon }}_{\mathrm{Az},k}}$ and $\sigma_{{\hat{\varepsilon }}_{\mathrm{El},k}}$ of the bearing angle ε=π/2−α, defined as the complementary of the Azimuth and Elevation angles, as shown in (10), where $M_{\mathrm{Az},k}$ and $M_{\mathrm{El},k}$ are the number of antenna elements, $SNR_{k}$ is the signal-to-noise ratio, $L_{\mathrm{Az},k}$ and $L_{\mathrm{El},k}$ are the total lengths of the array, *f* is the signal frequency, and *c* is the propagation speed of the electromagnetic wave.

(10) $$\left\{\begin{array}{c} \sigma_{{\hat{\varepsilon }}_{\mathrm{Az},k}}^{2}=\frac{12}{{\left(2\pi \right)}^{2}SNR_{k}\frac{\left(M_{\mathrm{Az},k}+1\right)}{\left(M_{\mathrm{Az},k}-1\right)}M_{\mathrm{Az},k}{\left(\frac{fL_{\mathrm{Az},k}}{c}\right)}^{2}\sin^{2}\left(\varepsilon_{\mathrm{Az},k}\right)}\;k=1,2,\ldots ,n\\ \sigma_{{\hat{\varepsilon }}_{\mathrm{El},k}}^{2}=\frac{12}{{\left(2\pi \right)}^{2}SNR_{k}\frac{\left(M_{\mathrm{El},k}+1\right)}{\left(M_{\mathrm{El},k}-1\right)}M_{\mathrm{El},k}{\left(\frac{fL_{\mathrm{El},k}}{c}\right)}^{2}\sin^{2}\left(\varepsilon_{\mathrm{El},k}\right)}\;k=1,2,\ldots ,n \end{array}\right.$$

Since the variance remains unchanged when adding a constant and is always positive, then $\sigma_{{\hat{\varepsilon }}_{\mathrm{Az},k}}^{2}=\sigma_{{\hat{\alpha }}_{\mathrm{Az},k}}^{2}$ and $\sigma_{{\hat{\varepsilon }}_{\mathrm{El},k}}^{2}=\sigma_{{\hat{\alpha }}_{\mathrm{El},k}}^{2}$.

In this study, among the assumptions regarding the measurement, it is considered that all the satellites are in target pointing, so $\varepsilon_{\mathrm{Az},1}=\varepsilon_{\mathrm{Az},2}=\cdots =\varepsilon_{\mathrm{Az},k}=\cdots =\varepsilon_{\mathrm{Az},n}=\pi /2$ and $\varepsilon_{\mathrm{El},1}=\varepsilon_{\mathrm{El},2}=\cdots =\varepsilon_{\mathrm{El},k}=\cdots =\varepsilon_{\mathrm{El},n}=\pi /2$. This hypothesis is related to attitude configuration and is considered because it leads to the desired and lowest value of the variance in (10) that can be achieved through the feedback commands of the Attitude Determination and Control System of the satellites. As a consequence, $\alpha_{\mathrm{Az},1}=\alpha_{\mathrm{Az},2}=\cdots =\alpha_{\mathrm{Az},k}=\cdots =\alpha_{\mathrm{Az},n}=0$ and $\alpha_{\mathrm{El},1}=\alpha_{\mathrm{El},2}=\cdots =\alpha_{\mathrm{El},k}=\cdots =\alpha_{\mathrm{El},n}=0$.

It is also assumed that every cross array of antennas of the satellite $S_{k}$ has the same length and the same number of elements in each direction, so $L_{\mathrm{Az},k}=L_{\mathrm{El},k}=L_{k}$ and $M_{\mathrm{Az},k}=M_{\mathrm{El},k}=M_{k}$.

Furthermore, it is assumed that every satellite has the same payload, so $L_{1}=L_{2}=\cdots =L_{k}=\cdots =L_{n}$ and $M_{1}=M_{2}=\cdots =M_{k}=\cdots =M_{n}$.

The consequence of these assumptions are that $\sigma_{{\hat{\alpha }}_{\mathrm{Az},1}}=\sigma_{{\hat{\alpha }}_{\mathrm{Az},2}}=\cdots =\sigma_{{\hat{\alpha }}_{\mathrm{Az},k}}=\cdots =\sigma_{{\hat{\alpha }}_{\mathrm{Az},n}}=\sigma_{{\hat{\alpha }}_{\mathrm{El},1}}=\sigma_{{\hat{\alpha }}_{\mathrm{El},2}}=\cdots =\sigma_{{\hat{\alpha }}_{\mathrm{El},k}}=\cdots =\sigma_{{\hat{\alpha }}_{\mathrm{El},n}}=\sigma$.

### 2.3. Dilution of Precision

The PDOP is one of the forms of dilution of precision DOP [35], which is the inverse of the square root of the trace of the matrix of the Fisher Information F, as shown in (11). In particular, in PDOP, the matrix F is a 3×3 matrix and is used to take into account the spatial distribution of the receivers in three dimensions.

(11) $$PDOP=\sqrt{Tr\left(F^{-1}\right)}$$

The Fisher Information Matrix can be decomposed as in (12), where C is the Covariance Matrix and H is the Geometric Design Matrix.

(12) $$F=H^{T}C^{-1}H$$

Under the assumptions of Gaussian, independent, and uncorrelated errors in the measurements, the Covariance Matrix C is diagonal, as shown in (13).

(13) $$C=\mathrm{diag}\left(\sigma_{{\hat{\alpha }}_{\mathrm{Az},1}}^{2},\sigma_{{\hat{\alpha }}_{\mathrm{El},1}}^{2},\sigma_{{\hat{\alpha }}_{\mathrm{Az},2}}^{2},\sigma_{{\hat{\alpha }}_{\mathrm{El},2}}^{2},\ldots ,\sigma_{{\hat{\alpha }}_{\mathrm{Az},k}}^{2},\sigma_{{\hat{\alpha }}_{\mathrm{El},k}}^{2},\ldots ,\sigma_{{\hat{\alpha }}_{\mathrm{Az},n}}^{2},\sigma_{{\hat{\alpha }}_{\mathrm{El},n}}^{2}\right)$$

Considering the assumptions made in Section 2.2, the Covariance Matrix C can be written as in (14), where $I_{2n}$ is the identity matrix of dimensions 2n×2n.

(14) $$C=\sigma^{2}I_{2n}$$

Following the approach in [36], the derivation of the Geometric Design Matrix H is composed by the set of derivatives of the equations of measurements, i.e., (9), with respect to the position coordinates of the emitter $\left(x_{E},y_{E},z_{E}\right)$, as shown in (15).

(15) $$H=\left[\begin{array}{ccc} \frac{\partial \alpha_{\mathrm{Az},1}}{\partial x_{E}} & \frac{\partial \alpha_{\mathrm{Az},1}}{\partial y_{E}} & \frac{\partial \alpha_{\mathrm{Az},1}}{\partial z_{E}}\\ \frac{\partial \alpha_{\mathrm{El},1}}{\partial x_{E}} & \frac{\partial \alpha_{\mathrm{El},1}}{\partial y_{E}} & \frac{\partial \alpha_{\mathrm{El},1}}{\partial z_{E}}\\ \frac{\partial \alpha_{\mathrm{Az},2}}{\partial x_{E}} & \frac{\partial \alpha_{\mathrm{Az},2}}{\partial y_{E}} & \frac{\partial \alpha_{\mathrm{Az},2}}{\partial z_{E}}\\ \frac{\partial \alpha_{\mathrm{El},2}}{\partial x_{E}} & \frac{\partial \alpha_{\mathrm{El},2}}{\partial y_{E}} & \frac{\partial \alpha_{\mathrm{El},2}}{\partial z_{E}}\\ \vdots & \vdots & \vdots \\ \frac{\partial \alpha_{\mathrm{Az},k}}{\partial x_{E}} & \frac{\partial \alpha_{\mathrm{Az},k}}{\partial y_{E}} & \frac{\partial \alpha_{\mathrm{Az},k}}{\partial z_{E}}\\ \frac{\partial \alpha_{\mathrm{El},k}}{\partial x_{E}} & \frac{\partial \alpha_{\mathrm{El},k}}{\partial y_{E}} & \frac{\partial \alpha_{\mathrm{El},k}}{\partial z_{E}}\\ \vdots & \vdots & \vdots \\ \frac{\partial \alpha_{\mathrm{Az},n}}{\partial x_{E}} & \frac{\partial \alpha_{\mathrm{Az},n}}{\partial y_{E}} & \frac{\partial \alpha_{\mathrm{Az},n}}{\partial z_{E}}\\ \frac{\partial \alpha_{\mathrm{El},n}}{\partial x_{E}} & \frac{\partial \alpha_{\mathrm{El},n}}{\partial y_{E}} & \frac{\partial \alpha_{\mathrm{El},n}}{\partial z_{E}} \end{array}\right]$$

In (16)–(18) are reported the derivatives of the Azimuth angle $\alpha_{\mathrm{Az},k}$ with respect to the variables $x_{E}$, $y_{E}$, and $z_{E}$.

(16) $$\begin{array}{c} \frac{\partial \alpha_{\mathrm{Az},k}}{\partial x_{E}}=\frac{\partial \left(\arctan\left(\frac{y_{E}-y_{S_{k}}}{x_{E}-x_{S_{k}}}\right)\right)}{\partial x_{E}}=\frac{-\left(y_{E}-y_{S_{k}}\right)}{{\left(x_{E}-x_{S_{k}}\right)}^{2}+{\left(y_{E}-y_{S_{k}}\right)}^{2}} \end{array}$$

(17) $$\begin{array}{c} \frac{\partial \alpha_{\mathrm{Az},k}}{\partial y_{E}}=\frac{\partial \left(\arctan\left(\frac{y_{E}-y_{S_{k}}}{x_{E}-x_{S_{k}}}\right)\right)}{\partial y_{E}}=\frac{\left(x_{E}-x_{S_{k}}\right)}{{\left(x_{E}-x_{S_{k}}\right)}^{2}+{\left(y_{E}-y_{S_{k}}\right)}^{2}} \end{array}$$

(18) $$\frac{\partial \alpha_{\mathrm{Az},k}}{\partial z_{E}}=\frac{\partial \left(\arctan\left(\frac{y_{E}-y_{S_{k}}}{x_{E}-x_{S_{k}}}\right)\right)}{\partial z_{E}}=0$$

In (19)–(21) are reported the derivatives of the Elevation angle $\alpha_{\mathrm{El},k}$ with respect to the variables $x_{E}$, $y_{E}$, and $z_{E}$.

(19) $$\begin{array}{c} \frac{\partial \alpha_{\mathrm{El},k}}{\partial x_{E}}=\frac{\partial \left(\arctan\left(\frac{\sqrt{{\left(x_{E}-x_{S_{k}}\right)}^{2}+{\left(y_{E}-y_{S_{k}}\right)}^{2}}}{z_{E}-z_{S_{k}}}\right)\right)}{\partial x_{\mathrm{El}},k}=\\ \frac{\left(z_{E}-z_{S_{k}}\right){\left({\left(x_{E}-x_{S_{k}}\right)}^{2}+{\left(y_{E}-y_{S_{k}}\right)}^{2}\right)}^{\frac{-1}{2}}\left(x_{E}-x_{S_{k}}\right)}{{\left(z_{E}-z_{S_{k}}\right)}^{2}+{\left(x_{E}-x_{S_{k}}\right)}^{2}+{\left(y_{E}-y_{S_{k}}\right)}^{2}} \end{array}$$

(20) $$\begin{array}{c} \frac{\partial \alpha_{\mathrm{El},k}}{\partial y_{E}}=\frac{\partial \left(\arctan\left(\frac{\sqrt{{\left(x_{E}-x_{S_{k}}\right)}^{2}+{\left(y_{E}-y_{S_{k}}\right)}^{2}}}{z_{E}-z_{S_{k}}}\right)\right)}{\partial y_{\mathrm{El}},k}=\\ \frac{\left(z_{E}-z_{S_{k}}\right){\left({\left(x_{E}-x_{S_{k}}\right)}^{2}+{\left(y_{E}-y_{S_{k}}\right)}^{2}\right)}^{\frac{-1}{2}}\left(y_{E}-y_{S_{k}}\right)}{{\left(z_{E}-z_{S_{k}}\right)}^{2}+{\left(x_{E}-x_{S_{k}}\right)}^{2}+{\left(y_{E}-y_{S_{k}}\right)}^{2}} \end{array}$$

(21) $$\begin{array}{c} \frac{\partial \alpha_{\mathrm{El},k}}{\partial z_{E}}=\frac{\partial \left(\arctan\left(\frac{\sqrt{{\left(x_{E}-x_{S_{k}}\right)}^{2}+{\left(y_{E}-y_{S_{k}}\right)}^{2}}}{z_{E}-z_{S_{k}}}\right)\right)}{\partial z_{\mathrm{El}},k}=\\ \frac{-\sqrt{{\left(x_{E}-x_{S_{k}}\right)}^{2}+{\left(y_{E}-y_{S_{k}}\right)}^{2}}}{{\left(z_{E}-z_{S_{k}}\right)}^{2}+{\left(x_{E}-x_{S_{k}}\right)}^{2}+{\left(y_{E}-y_{S_{k}}\right)}^{2}} \end{array}$$

Introducing the auxiliary quantities in (22) and (23) to compact the notation, the Geometric Design Matrix H is as in (24).

(22) $$s_{k}=\sqrt{{\left(x_{E}-x_{S,k}\right)}^{2}+{\left(y_{E}-y_{S,k}\right)}^{2}}$$

(23) $$u_{k}=\sqrt{{\left(x_{E}-x_{S,k}\right)}^{2}+{\left(y_{E}-y_{S,k}\right)}^{2}+{\left(z_{E}-z_{S,k}\right)}^{2}}$$

(24) $$H=\left[\begin{array}{ccc} -\frac{y_{E}-y_{S,1}}{s_{1}^{2}} & \frac{x_{E}-x_{S,1}}{s_{1}^{2}} & 0\\ -\frac{y_{E}-y_{S,2}}{s_{2}^{2}} & \frac{x_{E}-x_{S,2}}{s_{2}^{2}} & 0\\ \vdots & \vdots & \vdots \\ -\frac{y_{E}-y_{S,k}}{s_{k}^{2}} & \frac{x_{E}-x_{S,k}}{s_{k}^{2}} & 0\\ \vdots & \vdots & \vdots \\ -\frac{y_{E}-y_{S,n}}{s_{n}^{2}} & \frac{x_{E}-x_{S,n}}{s_{n}^{2}} & 0\\ \frac{\left(x_{E}-x_{S,1}\right)\left(z_{E}-z_{S,1}\right)}{s_{1}u_{1}^{2}} & \frac{\left(y_{E}-y_{S,1}\right)\left(z_{E}-z_{S,1}\right)}{s_{1}u_{1}^{2}} & -\frac{s_{1}}{u_{1}^{2}}\\ \frac{\left(x_{E}-x_{S,2}\right)\left(z_{E}-z_{S,2}\right)}{s_{2}u_{2}^{2}} & \frac{\left(y_{E}-y_{S,2}\right)\left(z_{E}-z_{S,2}\right)}{s_{2}u_{2}^{2}} & -\frac{s_{2}}{u_{2}^{2}}\\ \vdots & \vdots & \vdots \\ \frac{\left(x_{E}-x_{S,k}\right)\left(z_{E}-z_{S,k}\right)}{s_{k}u_{k}^{2}} & \frac{\left(y_{E}-y_{S,k}\right)\left(z_{E}-z_{S,k}\right)}{s_{k}u_{k}^{2}} & -\frac{s_{k}}{u_{k}^{2}}\\ \vdots & \vdots & \vdots \\ \frac{\left(x_{E}-x_{S,n}\right)\left(z_{E}-z_{S,n}\right)}{s_{n}u_{n}^{2}} & \frac{\left(y_{E}-y_{S,n}\right)\left(z_{E}-z_{S,n}\right)}{s_{n}u_{n}^{2}} & -\frac{s_{n}}{u_{n}^{2}} \end{array}\right]$$

Under the assumptions in (14), it is possible to simplify the expression in (12) as in (25).

(25) $$F=\frac{1}{\sigma^{2}}H^{T}H$$

Combining (25) and (11), (26) is obtained.

(26) $$PDOP=\sqrt{Tr\left(F^{-1}\right)}=\sigma \sqrt{Tr\left({\left(H^{T}H\right)}^{-1}\right)}$$

It is remarked that for some authors, especially in the GNSS literature (e.g., [37]), only the geometric part of (26) (i.e., the value of the square root that only depends on the Geometric Design Matrix H) is considered for the definition of the DOP, here indicated as $PDOP^{\prime }$, as shown in (27).

(27) $$PDOP^{\prime }=\frac{PDOP}{\sigma }=\sqrt{Tr\left({\left(H^{T}H\right)}^{-1}\right)}$$

The expression in (27) leads to the position error $\sigma_{p}$, which can be written [38] as shown in (28).

(28) $$\sigma_{p}=PDOP=PDOP^{\prime }\sigma$$

Since the purpose of this study is the minimization of the position error $\sigma_{p}$, and assuming that σ=const., the particular convention used for the definition of the DOP does not affect the result because $\min\left(\sigma_{p}\right)=\min\left(PDOP\right)=\min\left(PDOP^{\prime }\right)$.

One of the consequences of all the assumptions that leads to σ=const. is that the problem of minimization of PDOP (or $PDOP^{\prime }$) is independent of both the array geometry and the frequency of the signal.

The authors highlight that assuming σ=const. is a very strong hypothesis that comes from a high-level modeling of the system of satellites, and real-case applications must take into account the variations in the standard deviation σ, which depends on the actual fine design of the receiver and on SNR.

Another observation is that the value to be optimized considered in model (11) combined with the assumptions made so far in Section 2.1 and Section 2.2 is instantaneous and related to a single measurement per receiver.

### 2.4. Model of Orbits

In this study, a circular orbit is considered. The circle is a particular case of the ellipse, with the eccentricity *e* equal to 0: $e_{1}=e_{2}=\ldots =e_{k}=\ldots =e_{n}=0$. The semi-major axis *a* becomes equal to the radius of the orbit *r*: $a_{1}=r_{1}$, $a_{2}=r_{2}$, …, $a_{k}=r_{k}$, …, $a_{n}=r_{n}$.

Considering this assumption, (1) becomes as in (29).

(29) $${\left.x_{S_{k}}\right|}_{\mathrm{PQW}}=r_{k}\left(\begin{array}{c} \cos\left(\nu_{k}\right)\\ \sin\left(\nu_{k}\right)\\ 0 \end{array}\right)\;k=1,2,\ldots ,n$$

For a *k*-th satellite in a circular orbit, it is possible to define the height of a satellite as $h_{S_{k}}=r_{k}-r_{⊕}$.

Another consequence of the assumption of circular orbit is that the argument of the perigee is undefined: $\omega_{1}+\nu_{1}=\nu_{1}$, $\omega_{2}+\nu_{2}=\nu_{2}$, …, $\omega_{k}+\nu_{k}=\nu_{k}$, …, $\omega_{n}+\nu_{n}=\nu_{n}$; for some authors, it is common to denote this sum as “argument of latitude”, but, apparently, there is not a standard de facto letter to denote it.

This also means that the first matrix in (2) is an identity matrix: $R_{\left(\omega_{1},3\right)}=R_{\left(\omega_{2},3\right)}=\cdots =R_{\left(\omega_{k},3\right)}=\cdots =R_{\left(\omega_{n},3\right)}=I_{3}$.

Another assumption of this study is to consider an Earth scanning mission. This affects the value of the orbit inclination in considering a polar orbit: $i_{1}=i_{2}=\cdots =i_{k}=\cdots =i_{n}=\pi /2$. Therefore, the second matrix in (2) is given by (30) for every satellite.

(30) $$R_{\left(i_{k}=\frac{\pi }{2},1\right)}=\left[\begin{array}{ccc} 1 & 0 & 0\\ 0 & \cos\left(i_{k}=\frac{\pi }{2}\right) & -\sin\left(i_{k}=\frac{\pi }{2}\right)\\ 0 & \sin\left(i_{k}=\frac{\pi }{2}\right) & \cos\left(i_{k}=\frac{\pi }{2}\right) \end{array}\right]=\left[\begin{array}{ccc} 1 & 0 & 0\\ 0 & 0 & -1\\ 0 & 1 & 0 \end{array}\right],\;k=1,2,\ldots ,n$$

The inclination of the orbit of a satellite is one of the three angles that defines the orientation of the orbit plane into the space. An inclination of π/2 means that the satellite goes through the south–north direction and passes over the North and South Poles. This value of inclination combined with the rotation of the Earth guarantees that the satellite can scan all the surface of the Earth. It is pointed out that this value is chosen for a first-level evaluation, and actual values of the inclination are both mission-dependent (e.g., in some applications, the scans of some regions are not of interest) and epoch-dependent (i.e., the axis of rotation of the Earth is slightly inclined and changes over time).

Another assumption is to consider the same height of the orbits, so $r_{1}=r_{2}=\cdots =r_{k}=\cdots =r_{n}=r$ and $h_{S_{1}}=h_{S_{2}}=\cdots =h_{S_{k}}=\cdots =h_{S_{n}}=h_{S}$.

It can be recalled that the period of an orbit depends on the value of the radius of the orbit, so this assumptions guarantees that all the satellites have the same period.

The last hypothesis of this study considers a signal emitter on the Earth’s surface, so h=0 and (6) becomes as in (31).

(31) $$x_{E}{|}_{\mathrm{ECEF}}=\left(\begin{array}{c} r_{⊕}\cos\left(\Lambda \right)\cos\left(\lambda \right)\\ r_{⊕}\cos\left(\Lambda \right)\sin\left(\lambda \right)\\ r_{⊕}\sin\left(\Lambda \right) \end{array}\right)$$

### 2.5. Optimization Problem

From the assumptions made in Section 2.1–Section 2.4, the position dilution of precision PDOP has the functional dependence described in (32).

(32) $$\begin{array}{c} PDOP=f\left(x_{E},x_{S,1},x_{S,2},\ldots ,x_{S,k},\ldots ,x_{S,n}\right)\\ =f\left(\alpha_{\mathrm{GR}}\left(t\right),\Lambda ,\lambda ,\Omega_{1},\Omega_{2},\ldots ,\Omega_{k},\ldots ,\Omega_{n},\nu_{1},\nu_{2},\ldots ,\nu_{k},\ldots ,\nu_{n},r\right) \end{array}$$

In (32), convenient values of λ=0 and $\alpha_{\mathrm{GR}}=0$ can be assumed because from a relative perspective, there exists an equivalent shift in the values of the right ascension of the ascending nodes $\Omega_{1},\Omega_{2},\ldots ,\Omega_{k},\ldots ,\Omega_{n}$. This shift is the same for every satellite and is not relevant for the purposes of this study because it is focused on the relative geometry among satellites (i.e., differences in Ω). So, without loss of generalization, the functional dependence reported in (33) can be considered.

(33) $$PDOP=f\left(\Lambda ,\Omega_{1},\Omega_{2},\ldots ,\Omega_{k},\ldots ,\Omega_{n},\nu_{1},\nu_{2},\ldots ,\nu_{k},\ldots ,\nu_{n},r\right)$$

An optimization problem needs variables, domains, and constraints.

As previously investigated in [26], this kind of problem can be defined considering the right ascension of the ascending node $\Omega_{1},\Omega_{2},\ldots ,\Omega_{k},\ldots ,\Omega_{n}$ and the true anomaly $\nu_{1},\nu_{2},\ldots ,\nu_{k},\ldots ,\nu_{n}$ as a set of variables, while Λ and *r* are considered assigned parameters of a single instance of the problem. By definition, the domains of the right ascension of the ascending node and the true anomaly are $\Omega \in \left[0,2\pi \right]$ and $\nu \in \left[0,2\pi \right]$, so the whole set is $D={\left[0,2\pi \right]}^{2n}$.

Two main physical constraints are considered in this study. The first one is related to the fact that both the emitter and the satellites cannot be inside the surface of the Earth, so h≥0 and $r\geq r_{⊕}$ (equivalently, $h_{S}\geq 0$). The second condition is on the LOS. As derived in [26], indicating with $\Omega_{k,0}$ and $\nu_{k,0}$ the angular position for the *k*-th satellite corresponding to the Nadir direction with respect to the emitter position, there exists a maximum angle $\delta_{k}$ for the k-th satellite such that the condition on the satellite angles $\Omega_{k}$ and $\nu_{k}$ is expressed as in (34).

(34) $${\left(\Omega_{k}-\Omega_{k,0}\right)}^{2}+{\left(\nu_{k}-\nu_{k,0}\right)}^{2}\leq \delta_{k}^{2}$$

Thus, (34) is an absolute condition on the k-th satellite. However, considering also that the absolute angle $\delta_{k}$ can be expressed as

(35) $$\delta_{k}=\left(\frac{r_{⊕}}{r_{⊕}+h_{S_{k}}}\right)$$

and the assumption that all the satellites have the same orbit radius, the absolute condition (34) has the same maximum angle for all the satellites $\delta_{1}=\delta_{1}=\cdots =\delta_{k}=\cdots =\delta_{n}=\delta$.

As derived also in [26], (36) shows the problem of spatial optimization with the standard deviation on the position $\sigma_{p}$ as an objective function and $\Omega_{k,0}=\nu_{k,0}=0$ for every *k*-th satellite (i.e., the *k*-th LOS condition is written with respect to the Nadir alignment between the *k*-th satellite and the emitter).

(36) $$\left\{\begin{array}{c} \min_{\left(\Omega_{1},\Omega_{2},\ldots ,\Omega_{k},\ldots \Omega_{n},\nu_{1},\nu_{2},\ldots ,\nu_{k},\ldots ,\nu_{n}\right)\in D={\left[0,2\pi \right]}^{2n}}\left(\sigma_{p}\right)\\ s.t.\;\Omega_{1}^{2}+\nu_{1}^{2}\leq \delta^{2}\\ s.t.\;\Omega_{2}^{2}+\nu_{2}^{2}\leq \delta^{2}\\ \vdots \\ s.t.\;\Omega_{k}^{2}+\nu_{k}^{2}\leq \delta^{2}\\ \vdots \\ s.t.\;\Omega_{n}^{2}+\nu_{n}^{2}\leq \delta^{2} \end{array}\right.$$

## 3. Simulations

### 3.1. Two-Satellite Problem

In this study, simulations were conducted with two satellites, so the problem in (36) becomes as in (37).

(37) $$\left\{\begin{array}{c} \min_{\left(\Omega_{1},\Omega_{2},\nu_{1},\nu_{2}\right)\in {\left[0,2\pi \right]}^{4}}\left(\sigma_{p}\right)\\ s.t.\;\Omega_{1}^{2}+\nu_{1}^{2}\leq \delta^{2}\\ s.t.\;\Omega_{2}^{2}+\nu_{2}^{2}\leq \delta^{2} \end{array}\right.$$

Recalling the hypothesis of target pointing and the equation in (28), with a constant value of σ, representative of the fact that every satellite has the same receiver, the minimum of $\sigma_{p}$ occurs for the same set of variables for the minimum of PDOP.

As observed in [26], the problem in (37) can also be expressed in term of differences, as shown in (38). In particular, once the angular position of the first satellite is fixed, $\Omega_{1}$ and $\nu_{1}$, then the angular position of the second satellite, $\Omega_{2}$ and $\nu_{2}$, is calculated considering the difference with respect to the first satellite with $\Delta \Omega =\Omega_{2}-\Omega_{1}$ and $\Delta \nu =\nu_{2}-\nu_{1}$.

(38) $$\left\{\begin{array}{c} \min_{\left(\Delta \Omega ,\Delta \nu \right)\in {\left[0,2\pi \right]}^{2}}\left(\sigma_{p}\right)\\ s.t.\;\Delta \Omega^{2}+\Delta \nu^{2}\leq \delta_{1,2}^{2}\\ given\;\Omega_{1},\nu_{1} \end{array}\right.$$

The problem in (37) has four absolute variables $\left(\Omega_{1},\nu_{1},\Omega_{2},\nu_{2}\right)$ and two conditions, while the problem in (38) has two relative variables $\left(\Delta \Omega ,\Delta \nu \right)$ and one condition.

Considering the definition of ΔΩ and Δν, the relative condition in (38) ties together $\Omega_{1}$, $\nu_{1}$, $\Omega_{2}$, $\nu_{2}$, and $\delta_{1,2}$.

The problem in (38) can be considered a particular instance of the problem in (37) and has to be iterated varying the values of $\Omega_{1}$ and $\nu_{1}$ (and the parameters Λ and *r*), but it is important to remember firstly that neither of these angles must violate the LOS condition. It is also important to notice that $\delta_{1,2}$ is a relative condition of the satellite $S_{2}$ with respect to satellite $S_{1}$.

In this study, a conservative the relationship among the absolute angle δ and its relative $\delta_{1,2}$ is expressed as in (39).

(39) $$\delta \geq \delta_{1,2}+\sqrt{{\left(\max|\Omega_{1}|\right)}^{2}+{\left(\max|\nu_{1}|\right)}^{2}}$$

The meaning of (39) is that the maximum displacement of the first satellite $S_{1}$ plus the relative displacement of the second satellite $S_{2}$ with respect to the first one, expressed by $\delta_{1,2}$, must be lower than or equal to the absolute angular condition δ.

A convenient hypothesis, which is more conservative for simulation purposes, is to consider $\max|\Omega_{1}\left|=\max\right|\nu_{1}|=\delta_{1,2}=\delta_{r}$.

With this assumption, the condition (39) becomes as in (40).

(40) $$\delta \geq \delta_{r}+\sqrt{\delta_{r}^{2}+\delta_{r}^{2}}=\delta_{r}+\sqrt{2\delta_{r}^{2}}=\delta_{r}+\sqrt{2}\delta_{r}=\left(1+\sqrt{2}\right)\delta_{r}$$

The condition (40) can also be expressed as in (41).

(41) $$\delta_{r}\leq \frac{\delta }{1+\sqrt{2}}$$

From (41), the ranges for $\Omega_{1}$ and $\nu_{1}$ in which the problem in (38) has to be iterated become as in (42) and guarantee the LOS condition in the case of $\Omega_{1,0}=\nu_{1,0}=0$ (i.e., the LOS condition is written with respect to the Nadir alignment between the first satellite and the emitter).

(42) $$\begin{array}{c} \frac{-\delta }{1+\sqrt{2}}\leq \Omega_{1}\leq \frac{\delta }{1+\sqrt{2}}\\ \frac{-\delta }{1+\sqrt{2}}\leq \nu_{1}\leq \frac{\delta }{1+\sqrt{2}} \end{array}$$

To generalize the problem and relax the limitations imposed by $\Omega_{1,0}=0$ and $\nu_{1,0}=0$, a further consideration can be made of their meaning. It must be observed that the values of $\Omega_{1}$ and $\nu_{1}$ are with respect to the value of the latitude Λ and longitude λ of the emitter. In general, the range of interest should be centered on the Nadir direction with respect to the emitter position, so their values have to be corrected considering $\Omega_{1}\to \Omega_{1}+\lambda$ and $\nu_{1}\to \nu_{1}+\Lambda$; then, (42) becomes as in (43).

(43) $$\begin{array}{c} \frac{-\delta }{1+\sqrt{2}}\leq \Omega_{1}+\lambda \leq \frac{\delta }{1+\sqrt{2}}\\ \frac{-\delta }{1+\sqrt{2}}\leq \nu_{1}+\Lambda \leq \frac{\delta }{1+\sqrt{2}} \end{array}$$

An important observation is that this way of reasoning, which permits one to pass directly from the conditions written in terms of $\Omega_{1,0}$ and $\nu_{1,0}$ to Λ and λ, is always valid for a spherical Earth. When an ellipsoidal model is used, the condition on the right ascension of the ascending node $\Omega_{1}$ in (43) is still valid in any case, but the condition on the true anomaly $\nu_{1}$ needs special attention because of the distinction between the geodetic latitude and geocentric latitude.

Since a variation of the results with respect to the value of the longitude λ is not expected (i.e., because a variation in longitude can be compensated for by a shift on the right ascension of the ascending node, obtaining the same results), in this study, it is assumed that λ=0.

The value of λ≠0 on the conditions in (43) can take place as theoretical completeness or can be of interest in simulators, where the current value of $\alpha_{\mathrm{GR}}\left(t\right)$ in a certain time epoch *t* is of interest.

On the other hand, the variation of results with respect to the latitude Λ is of interest because with an inclination of π/2, the trajectories of the orbits tend to converge to the same point in the poles of the Earth.

Another angle considered among the results of this study is the spacing angle $\gamma_{1,2}=∠S_{1}ES_{2}$, which can be calculated as in (44). This angle represents the spacing between the satellites $S_{1}$ and $S_{2}$ with respect to the position of the emitter *E*.

(44) $$\gamma_{1,2}=\left(\frac{\vec{ES_{1}}}{∥\vec{ES_{1}}∥}\cdot \frac{\vec{ES_{2}}}{∥\vec{ES_{2}}∥}\right)$$

### 3.2. Simulation Settings and Results

Simulations of the problem in (38) with the conditions expressed in (39) and (43) were conducted in the MATLAB 2024a environment with the aid of the Symbolic Math Toolbox and the Optimization Toolbox: the first was used to write the optimization problem on the objective function (i.e., the PDOP) in terms of symbolic variables, the latter was used to solve the problem written in terms of symbolic variables and obtain the optimal solution (i.e., the set of angles of the right ascension of the ascending node and the true anomaly for each satellite).

To solve the problem, the interior point algorithm was chosen; this was because it is mathematically proven that it is able to effectively solve a nonlinear objective function subject to nonlinear inequality constraints converging to an approximate solution defined by the user-defined tolerances; furthermore, it always satisfies the boundary conditions at all iterations and can recover from possible NaN (i.e., not a number) or Inf (i.e., infinite) results in every iteration. As expected, from the conducted campaign of simulations, the algorithm was demonstrated to be always convergent. Mathematical details of the algorithm can be found in [39].

In particular, considering x as the solution to the problem, $g\left(x\right)$ as the nonlinear objective function, $w_{1}\left(x\right)\leq 0,w_{2}\left(x\right)\leq 0,\ldots ,w_{l}\left(x\right)\leq 0,w_{m}\left(x\right)\leq 0$ as the *m* nonlinear inequality constraint conditions, and $x_{j}$ as the solution calculated at *j*-th iteration, the tolerances are defined in relative terms: the condition on step tolerance is defined as shown in (45), where $T_{s}>0$ is the step tolerance; the optimality condition is defined as shown in (46), where $T_{o}>0$ is the optimality tolerance, $\lambda_{1}\geq 0,\lambda_{2}\geq 0,\ldots ,\lambda_{l},\ldots ,\lambda_{m}\geq 0$ are the Lagrange multipliers, and ${∥\cdot ∥}_{\infty }$ denotes the infinity norm; the constraint condition is defined as shown in (47), where $T_{c}>0$ is the constraint tolerance.

(45) $$∥x_{j}-x_{j+1}∥<T_{s}\left(1+∥x_{j}∥\right)$$

(46) $$∥\nabla g\left(x_{j+1}\right)+\sum_{l=1}^{m}\left(\lambda_{l}w_{l}\left(x_{j+1}\right)\right)∥<T_{o}{∥\nabla g\left(x_{j+1}\right)∥}_{\infty }$$

(47) $$\left\{\begin{array}{c} ∥w_{1}\left(x_{j}\right)∥<T_{c}\\ ∥w_{2}\left(x_{j}\right)∥<T_{c}\\ \vdots \\ ∥w_{l}\left(x_{j}\right)∥<T_{c}\\ \vdots \\ ∥w_{m}\left(x_{j}\right)∥<T_{c} \end{array}\right.$$

It can be recalled that the left hand of (46) is a particular case of the more general Lagrangian of the Karush–Kuhn–Tucker (KKT) conditions [40], which considers both equality and inequality constraints for convex and nonconvex problems; as can be seen from (38), the optimization problem that this study aims to solve involves only inequality constraints.

The iterations are stopped on a certain fixed number (e.g., the default value is 1000) when one of the conditions on step tolerance (45) (i.e., the relative difference in values) or optimality tolerance (46) (that is, the values of the first-order derivatives, KKT condition) is met; in this latter case, the constraint tolerance (47) is always satisfied.

Details on the numerical implementation of the optimization algorithm, stopping criteria, and derivation of formulas used for the calculation of tolerances can be found in the MATLAB User Guide for the Optimization Toolbox [41] and the dedicated literature.

For this algorithm, the following settings were considered for the iterations of the solver: a step tolerance of $T_{s}=10^{-15}$, an optimality tolerance of $T_{o}=10^{-12}$, a constraint tolerance of $T_{c}=10^{-12}$, an interior point algorithm, a scheme of central finite differences, and a maximum number of iterations equal to 2400.

The value of the maximum number of iterations was chosen in order to never be reached.

The choice of the settings was made observing the precision of the solution. In particular, the selected case for these tests was the one with the emitter position in Λ=30°, λ=0°, and h=0 m. The altitudes of the satellites were $h_{S}=400$ km and the angular position of the first satellite was $\Omega_{1}=\left.\delta \left(h_{S}\right)/\left(1+\sqrt{2}\right)\right.\approx 8.198361918$°, $\nu_{1}=\Lambda -\left.\delta \left(h_{S}\right)/\left(1+\sqrt{2}\right)\right.\approx 21.80163808$°. The choice of the first satellite at the border of the constraints was made to stress the solver not only on the step tolerance $T_{s}$ and the optimality tolerance $T_{o}$ but also on the tolerance related to the constraints $T_{c}$.

The selected cases of the tests were on step tolerance $T_{s}$, $T_{o}$, and $T_{c}$ from $10^{-3}$ to $10^{-12}$. The results of the tests were for the precision of the numerical figures of the solution and are reported in Table 1. For simulations, the step tolerance was further decreased to $T_{s}=10^{-15}$ because it was observed that this was the main reason that the convergence of the solver stopped.

Even if the numerical convergence analysis was conducted for up to 15 figures and beyond, the solutions needed to be considered physically valid for up to 7 figures because the limitation was on the input data: in particular, the mean Earth radius $r_{⊕}$ considered in this study was precise for up to 7 figures.

In particular, 17 evenly spaced values of the latitude of the emitter Λ from −80° to 80° and 9 evenly spaced values of the orbit radius *r* with *h* from 400 km to 2000 km were considered; 14 evenly spaced values were considered for both $\Omega_{1}$ and $\nu_{1}$.

Since, as observed in a previous work [26], in this kind of problem, multiple values of local minimum occur, a pre-evaluation of the objective function with a fine grid of 0.01° × 0.01° was considered for the selection of the initial value of the solver.

The total number of simulated cases was 29,988. In all simulated cases, the constraints were satisfied and the solution existed. In 28,798 cases, the solver stopped on the condition on the step tolerance, while, in 1190 cases, it stopped on the optimality tolerance. The maximum number of iterations was never met.

**Figure 1:**
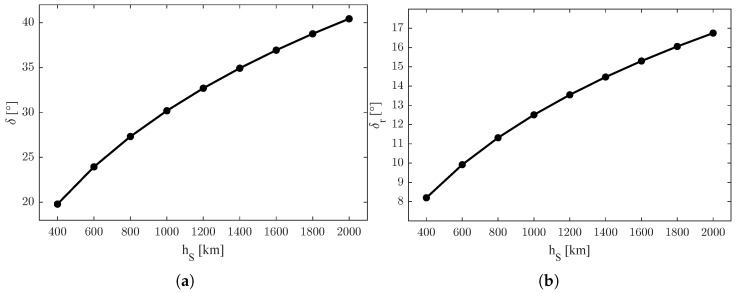
Representation of the values of LOS constraints. (**a**) Values of the absolute constraint angle δ with respect to the orbit height of the satellites $h_{S}$. (**b**) Values of the relative constraint angle $\delta_{r}$ with respect to the orbit height of the satellites $h_{S}$.

Figure 1a shows the values of the absolute constraint angle δ and Figure 1b the relative constraint angle $\delta_{r}$ with respect to the orbit height of the satellites $h_{S}$. In particular, it can be noticed that the constraint angle is tighter with lower heights of the orbit.

The values of

(48) $$\begin{array}{c} \Delta \Omega =\Delta \Omega \left(\Omega_{1},\nu_{1},\Lambda ,r\right)\\ \Delta \nu =\Delta \nu \left(\Omega_{1},\nu_{1},\Lambda ,r\right)\\ \gamma_{1,2}=\gamma_{1,2}\left(\Omega_{1},\nu_{1},\Lambda ,r\right) \end{array}$$

are the main results of this study. ΔΩ and Δν are the optima, while $\gamma_{1,2}$ is the spacing angle between the satellites with respect to the emitter. Since they are 4D functions, the number of necessary graphical representations on the 2D plane for each function was 6.

To take into account that in some representation planes, the results are in different ranges with respect to $\nu_{1}$ because a shift in the latitude of the emitter requires a value of $\nu_{1,0}=\Lambda$ for Nadir alignment, the independent variable $\nu_{1}$ was replaced with $\nu_{1}^{\Lambda }=\left(\nu_{1}-\nu_{1,0}\right)=\left(\nu_{1}-\Lambda \right)$, as shown in (49)–(51).

(49) $$\Delta \Omega =\Delta \Omega \left(\Omega_{1},\nu_{1}-\nu_{1,0},\Lambda ,r\right)=\Delta \Omega \left(\Omega_{1},\nu_{1}-\Lambda ,\Lambda ,r\right)=\Delta \Omega \left(\Omega_{1},\nu_{1}^{\Lambda },\Lambda ,r\right)$$

(50) $$\Delta \nu =\Delta \nu \left(\Omega_{1},\nu_{1}-\nu_{1,0},\Lambda ,r\right)=\Delta \nu \left(\Omega_{1},\nu_{1}-\Lambda ,\Lambda ,r\right)=\Delta \nu \left(\Omega_{1},\nu_{1}^{\Lambda },\Lambda ,r\right)$$

(51) $$\gamma_{1,2}=\gamma_{1,2}\left(\Omega_{1},\nu_{1}-\nu_{1,0},\Lambda ,r\right)=\gamma_{1,2}\left(\Omega_{1},\nu_{1}-\Lambda ,\Lambda ,r\right)=\Delta \gamma_{1,2}\left(\Omega_{1},\nu_{1}^{\Lambda },\Lambda ,r\right)$$

**Figure 2:**
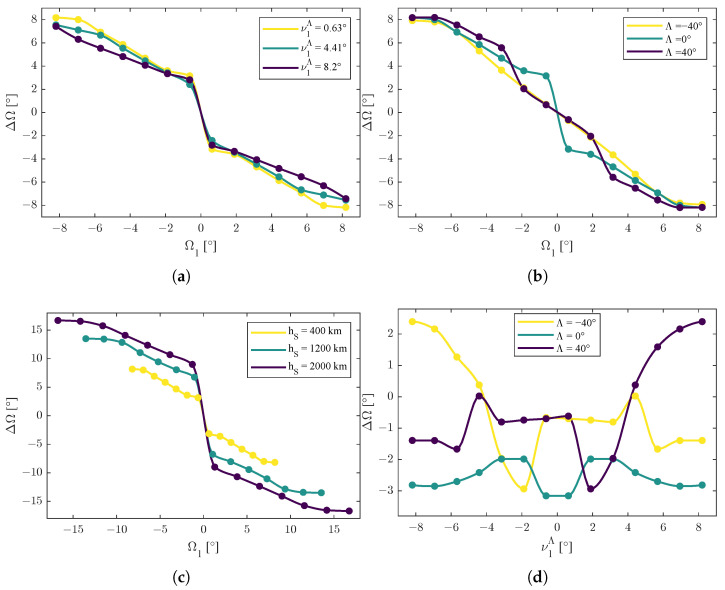

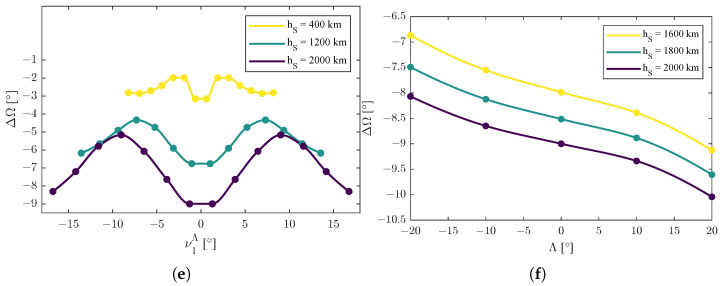
Trends of the function $\Delta \Omega =\Delta \Omega \left(\Omega_{1},\nu_{1}^{\Lambda },\Lambda ,r\right)$, representative of optima of the differences between the values of the right ascension of the ascending node of the two satellites. Dots are the results of the simulation; lines are interpolated values. (**a**) Graphic representation of the function $\Delta \Omega =\Delta \Omega \left(\Omega_{1},\nu_{1}^{\Lambda },\Lambda_{*},r_{*}\right)$. (**b**) Graphic representation of the function $\Delta \Omega =\Delta \Omega \left(\Omega_{1},\nu_{1,*}^{\Lambda },\Lambda ,r_{*}\right)$. (**c**) Graphic representation of the function $\Delta \Omega =\Delta \Omega \left(\Omega_{1},\nu_{1,*}^{\Lambda },\Lambda_{*},r\right)$. (**d**) Graphic representation of the function $\Delta \Omega =\Delta \Omega \left(\Omega_{1,*},\nu_{1}^{\Lambda },\Lambda ,r_{*}\right)$. (**e**) Graphic representation of the function $\Delta \Omega =\Delta \Omega \left(\Omega_{1,*},\nu_{1}^{\Lambda },\Lambda_{*},r\right)$. (**f**) Graphic representation of the function $\Delta \Omega =\Delta \Omega \left(\Omega_{1,*},\nu_{1,*}^{\Lambda },\Lambda ,r\right)$.

**Figure 3:**
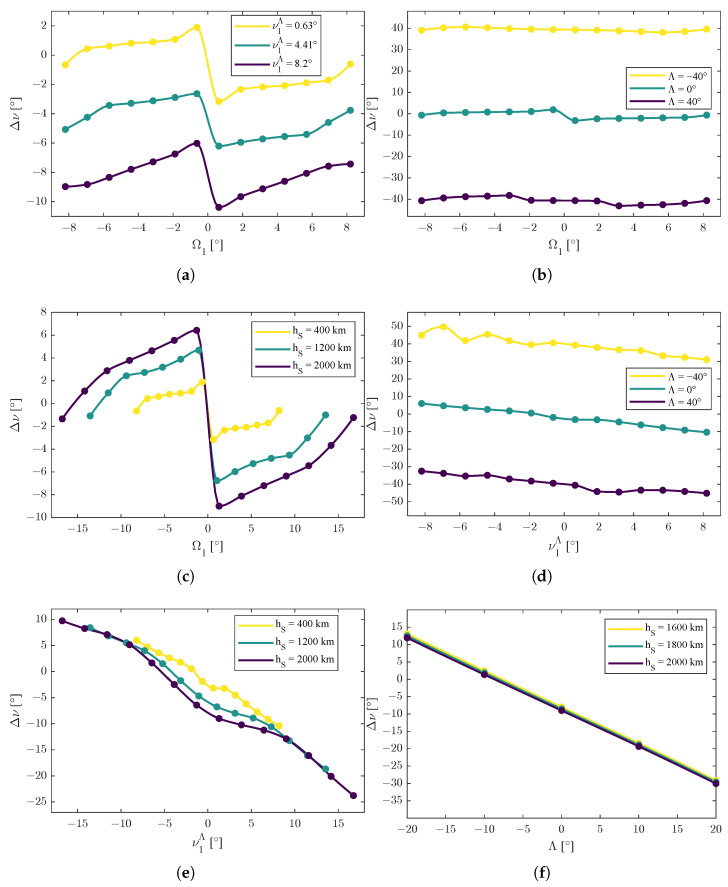
Trends of the function $\Delta \nu =\Delta \nu \left(\Omega_{1},\nu_{1}^{\Lambda },\Lambda ,r\right)$, representative of optima of the differences between the values of the true anomaly of the two satellites. Dots are the results of the simulation; lines are interpolated values. (**a**) Graphic representation of the function $\Delta \nu =\Delta \nu \left(\Omega_{1},\nu_{1}^{\Lambda },\Lambda_{*},r_{*}\right)$. (**b**) Graphic representation of the function $\Delta \nu =\Delta \nu \left(\Omega_{1},\nu_{1,*}^{\Lambda },\Lambda ,r_{*}\right)$. (**c**) Graphic representation of the function $\Delta \nu =\Delta \nu \left(\Omega_{1},\nu_{1,*}^{\Lambda },\Lambda_{*},r\right)$. (**d**) Graphic representation of the function $\Delta \nu =\Delta \nu \left(\Omega_{1,*},\nu_{1}^{\Lambda },\Lambda ,r_{*}\right)$. (**e**) Graphic representation of the function $\Delta \nu =\Delta \nu \left(\Omega_{1,*},\nu_{1}^{\Lambda },\Lambda_{*},r\right)$. (**f**) Graphic representation of the function $\Delta \nu =\Delta \nu \left(\Omega_{1,*},\nu_{1,*}^{\Lambda },\Lambda ,r\right)$.

**Figure 4:**
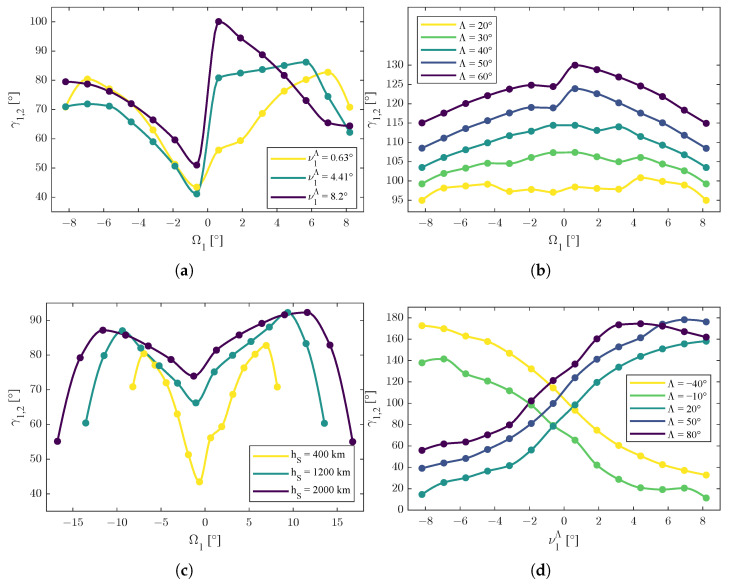

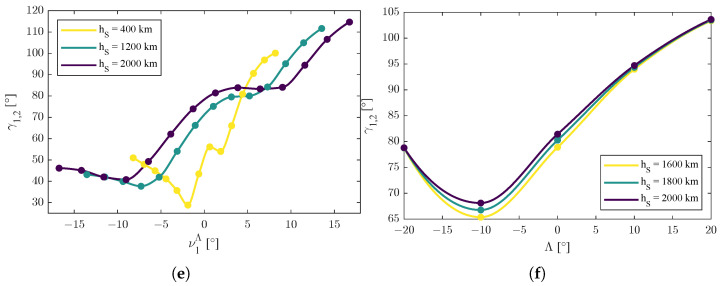
Trends of the function $\gamma_{1,2}=\gamma_{1,2}\left(\Omega_{1},\nu_{1}^{\Lambda },\Lambda ,r\right)$, representative of optima of the values of the spacing angle between the two satellites. Dots are the results of the simulation; lines are interpolated values. (**a**) Graphic representation of the function $\gamma_{1,2}=\gamma_{1,2}\left(\Omega_{1},\nu_{1}^{\Lambda },\Lambda_{*},r_{*}\right)$. (**b**) Graphic representation of the function $\gamma_{1,2}=\gamma_{1,2}\left(\Omega_{1},\nu_{1,*}^{\Lambda },\Lambda ,r_{*}\right)$. (**c**) Graphic representation of the function $\gamma_{1,2}=\gamma_{1,2}\left(\Omega_{1},\nu_{1,*}^{\Lambda },\Lambda_{*},r\right)$. (**d**) Graphic representation of the function $\gamma_{1,2}=\gamma_{1,2}\left(\Omega_{1,*},\nu_{1}^{\Lambda },\Lambda ,r_{*}\right)$. (**e**) Graphic representation of the function $\gamma_{1,2}=\gamma_{1,2}\left(\Omega_{1,*},\nu_{1}^{\Lambda },\Lambda_{*},r\right)$. (**f**) Graphic representation of the function $\gamma_{1,2}=\gamma_{1,2}\left(\Omega_{1,*},\nu_{1,*}^{\Lambda },\Lambda ,r\right)$.

In Figure 2, Figure 3 and Figure 4, the dots are the calculated values obtained by the solver. Among them, the trend lines were obtained through an interpolation with a Piecewise Cubic Hermite Interpolating Polynomial of 100 points for each curve [42]: the choice of this interpolation method was made for graphical purposes and was due to its shape-preserving properties and to avoid as much as possible unwanted oscillations among the values.

In particular, for the function $\Delta \Omega =\Delta \Omega \left(\Omega_{1},\nu_{1}^{\Lambda },\Lambda ,r\right)$, the following functions

(52) $$\begin{array}{c} \Delta \Omega =\Delta \Omega \left(\Omega_{1},\nu_{1}^{\Lambda },\Lambda_{*},r_{*}\right)\\ \Delta \Omega =\Delta \Omega \left(\Omega_{1},\nu_{1,*}^{\Lambda },\Lambda ,r_{*}\right)\\ \Delta \Omega =\Delta \Omega \left(\Omega_{1},\nu_{1,*}^{\Lambda },\Lambda_{*},r\right)\\ \Delta \Omega =\Delta \Omega \left(\Omega_{1,*},\nu_{1}^{\Lambda },\Lambda ,r_{*}\right)\\ \Delta \Omega =\Delta \Omega \left(\Omega_{1,*},\nu_{1}^{\Lambda },\Lambda_{*},r\right)\\ \Delta \Omega =\Delta \Omega \left(\Omega_{1,*},\nu_{1,*}^{\Lambda },\Lambda ,r\right) \end{array}$$

were considered for the representations in Figure 2, where $\Omega_{1,*}$, $\nu_{1,*}^{\Lambda }$, $\Lambda_{*}$, and $r_{*}$ are fixed values.

For representations of trends, $\Lambda_{*}=0$° was chosen, and $r_{*}$ corresponded to the height $h_{S_{*}}=400$ km.

Because of a jump discontinuity in the resulting trends for $\Omega_{1}=0$ and $\nu_{1}^{\Lambda }=0$, these values were not chosen for $\Omega_{1,*}$ and $\nu_{1,*}^{\Lambda }$. Instead, for $\Omega_{1,*}$ and $\nu_{1,*}^{\Lambda }$, the first positive values in the range of simulated values were considered.

These first positive values depended on how the ranges for $\Omega_{1}$ and $\nu_{1}^{\Lambda }$ were defined and spaced in the simulation environment; in this study, 14 evenly spaced values were considered, but the ranges depended on the relative condition $\delta_{r}$. Considering these two facts, the first positive values for $\Omega_{1,*}$ and $\nu_{1,*}^{\Lambda }$ depended on the value of the orbit radius *r*: it varied from ≈0.63° for $h_{S_{*}}=400$ km to ≈1.29° for $h_{S}=2000$ km.

For the function $\Delta \nu =\Delta \nu \left(\Omega_{1},\nu_{1}^{\Lambda },\Lambda ,r\right)$, the following functions

(53) $$\begin{array}{c} \Delta \nu =\Delta \nu \left(\Omega_{1},\nu_{1}^{\Lambda },\Lambda_{*},r_{*}\right)\\ \Delta \nu =\Delta \nu \left(\Omega_{1},\nu_{1,*}^{\Lambda },\Lambda ,r_{*}\right)\\ \Delta \nu =\Delta \nu \left(\Omega_{1},\nu_{1,*}^{\Lambda },\Lambda_{*},r\right)\\ \Delta \nu =\Delta \nu \left(\Omega_{1,*},\nu_{1}^{\Lambda },\Lambda ,r_{*}\right)\\ \Delta \nu =\Delta \nu \left(\Omega_{1,*},\nu_{1}^{\Lambda },\Lambda_{*},r\right)\\ \Delta \nu =\Delta \nu \left(\Omega_{1,*},\nu_{1,*}^{\Lambda },\Lambda ,r\right) \end{array}$$

were considered for the representations in Figure 3. The same considerations made for ΔΩ applied for the values of $\Omega_{1,*}$, $\nu_{1,*}^{\Lambda }$, $\Lambda_{*}$, and $r_{*}$ in the representation of the Δν function.

For the function $\gamma_{1,2}=\gamma_{1,2}\left(\Omega_{1},\nu_{1}^{\Lambda },\Lambda ,r\right)$, the following functions

(54) $$\begin{array}{c} \gamma_{1,2}=\gamma_{1,2}\left(\Omega_{1},\nu_{1}^{\Lambda },\Lambda_{*},r_{*}\right)\\ \gamma_{1,2}=\gamma_{1,2}\left(\Omega_{1},\nu_{1,*}^{\Lambda },\Lambda ,r_{*}\right)\\ \gamma_{1,2}=\gamma_{1,2}\left(\Omega_{1},\nu_{1,*}^{\Lambda },\Lambda_{*},r\right)\\ \gamma_{1,2}=\gamma_{1,2}\left(\Omega_{1,*},\nu_{1}^{\Lambda },\Lambda ,r_{*}\right)\\ \gamma_{1,2}=\gamma_{1,2}\left(\Omega_{1,*},\nu_{1}^{\Lambda },\Lambda_{*},r\right)\\ \gamma_{1,2}=\gamma_{1,2}\left(\Omega_{1,*},\nu_{1,*}^{\Lambda },\Lambda ,r\right) \end{array}$$

were considered for the representations in Figure 4. The same considerations made for ΔΩ applied for the values of $\Omega_{1,*}$, $\nu_{1,*}^{\Lambda }$, $\Lambda_{*}$, and $r_{*}$ in the representation of the $\gamma_{1,2}$ function.

### 3.3. Analysis of Results

This subsection reports the interpretation of the results in Section 3.2.

The first set of functions were the ones in (52). From Figure 2a–c, the dependence of ΔΩ with respect to the right ascension of the ascending node of the first satellite $\Omega_{1}$ revealed that it was always decreasing, with a possible presence of a jump discontinuity in the value of $\Omega_{1}=0$.

Furthermore, ΔΩ appeared also to be anti-symmetric with respect to $\Omega_{1}$, i.e., $\Delta \Omega \left(\Omega_{1}\right)=-\Delta \Omega \left(-\Omega_{1}\right)$. This behavior confirms the prediction of a previous investigation [26], even if it was restricted to a selected instance of the problem.

A possible approximate and linear model for ΔΩ with respect to $\Omega_{1}$ to capture the behaviors of these results is

(55) $$\begin{array}{c} \Delta \Omega \left(\Omega_{1},\nu_{1}^{\Lambda },\Lambda_{*},r_{*}\right)\approx -k_{\nu_{1},1}\left(\nu_{1}^{\Lambda },\Lambda_{*},r_{*}\right)\Omega_{1}\\ +k_{\nu_{1},2}\left(\nu_{1}^{\Lambda },\Lambda_{*},r_{*}\right)\mathrm{sgn}\left(\Omega_{1}\right)+f_{\nu_{1}}\left(\nu_{1}^{\Lambda },\Lambda_{*},r_{*}\right) \end{array}$$

with $k_{\nu_{1},1}\left(\nu_{1}^{\Lambda },\Lambda_{*},r_{*}\right),k_{\nu_{1},2}\left(\nu_{1}^{\Lambda },\Lambda_{*},r_{*}\right)\in R_{>0}$ as linear coefficients and $f_{\nu_{1}}\left(\nu_{1}^{\Lambda },\Lambda_{*},r_{*}\right)$ as a nonlinear function with minimal effect,

(56) $$\begin{array}{c} \Delta \Omega \left(\Omega_{1},\nu_{1,*}^{\Lambda },\Lambda ,r_{*}\right)\approx -k_{\Lambda ,1}\left(\nu_{1,*}^{\Lambda },\Lambda ,r_{*}\right)\Omega_{1}\\ +k_{\Lambda ,2}\left(\nu_{1,*}^{\Lambda },\Lambda ,r_{*}\right)\mathrm{sgn}\left(\Omega_{1}\right)+f_{\Lambda }\left(\nu_{1,*}^{\Lambda },\Lambda ,r_{*}\right) \end{array}$$

with $k_{\Lambda ,1}\left(\nu_{1,*}^{\Lambda },\Lambda ,r_{*}\right),k_{\Lambda ,2}\left(\nu_{1,*}^{\Lambda },\Lambda ,r_{*}\right)\in R_{>0}$ as linear coefficients and $f_{\Lambda }\left(\nu_{1,*}^{\Lambda },\Lambda ,r_{*}\right)$ as a nonlinear function with minimal effect, and

(57) $$\begin{array}{c} \Delta \Omega \left(\Omega_{1},\nu_{1,*}^{\Lambda },\Lambda_{*},r\right)\approx -k_{r,1}\left(\nu_{1,*}^{\Lambda },\Lambda_{*},r\right)\Omega_{1}\\ +k_{r,2}\left(\nu_{1,*}^{\Lambda },\Lambda_{*},r\right)\mathrm{sgn}\left(\Omega_{1}\right)+f_{r}\left(\nu_{1,*}^{\Lambda },\Lambda_{*},r\right) \end{array}$$

with $k_{r,1}\left(\nu_{1,*}^{\Lambda },\Lambda_{*},r\right),k_{r,2}\left(\nu_{1,*}^{\Lambda },\Lambda_{*},r\right)\in R_{>0}$ as linear coefficients and $f_{r}\left(\nu_{1,*}^{\Lambda },\Lambda_{*},r\right)$ as a nonlinear function with minimal effect.

In Figure 2c, it can be noticed also that the function ΔΩ with respect to the orbit radius *r* presented an amplification and a change of scale with respect to $\Omega_{1}$, so

(58) $$\Delta \Omega \left(\Omega_{1},\nu_{1,*}^{\Lambda },\Lambda_{*},r\right)\approx -k_{1}\left(r\right)\Delta \Omega \left(-k_{2}\left(r\right)\Omega_{1},\nu_{1,*}^{\Lambda },\Lambda_{*}\right)$$

could also be an alternative possible model with $k_{1}\left(r\right),k_{2}\left(r\right)\in R\setminus \left\{0\right\}$.

From Figure 2a,d,e, the dependence of ΔΩ with respect to $\nu_{1}^{\Lambda }$ appears more complicated. In selected cases of [26], a symmetric behavior such as $\Delta \Omega \left(\nu_{1}^{\Lambda }\right)=\Delta \Omega \left(-\nu_{1}^{\Lambda }\right)$ was predicted, but the extended analysis of the present study reveals that $\Delta \Omega \left(\nu_{1}^{\Lambda }\right)$ is still symmetric when varying the orbit radius *r* (i.e., $h_{S}$)) but has more complicated behavior with respect to the latitude of the emitter when Λ≠0, revealing a local minimum and a local maximum, as shown in Figure 2d.

As shown in Figure 2f, there was a decreasing behavior with respect to the orbit radius *r* (i.e., the height $h_{S}$). This is also evident in Figure 2e and for positive values of $\Omega_{1}$ in Figure 2c, so

(59) $$\begin{array}{cc} \frac{\partial \left(\Delta \Omega \right)}{\partial r}>0 & \Omega_{1}>0\\ \frac{\partial \left(\Delta \Omega \right)}{\partial r}<0 & \Omega_{1}<0 \end{array}$$

is a possible interpretation of the data.

The second set of functions were the ones reported in (53). From Figure 3a–c, the dependence of Δν with respect to $\Omega_{1}$ reveals an anti-symmetric behavior, i.e., $\Delta \nu \left(\Omega_{1}\right)=-\Delta \nu \left(-\Omega_{1}\right)$.

In addition to anti-symmetry with respect to $\Omega_{1}$, there was a translation with respect to $\nu_{1}^{\Lambda }$, i.e.,

(60) $$\Delta \nu \left(\Omega_{1},\nu_{1}^{\Lambda },\Lambda_{*},r_{*}\right)\approx -\Delta \nu \left(-\Omega_{1},\nu_{1}^{\Lambda },\Lambda_{*},r_{*}\right)+k\left(\nu_{1}^{\Lambda }\right)\mathrm{sgn}\left(\nu_{1}^{\Lambda }\right)$$

with $k\left(\nu_{1}^{\Lambda }\right)\in R_{>0}$; the sign function modeled the mathematical jump.

There was a translation also with respect to Λ, i.e.,

(61) $$\Delta \nu \left(\Omega_{1},\nu_{1,*}^{\Lambda },\Lambda ,r_{*}\right)\approx -\Delta \nu \left(-\Omega_{1},\nu_{1,*}^{\Lambda },\Lambda ,r_{*}\right)+k\left(\Lambda \right)\mathrm{sgn}\left(\Lambda \right)$$

with $k\left(\Lambda \right)\in R_{>0}$.

With respect to the orbit radius *r*, the function Δν presented an amplification and a change of scale with respect to $\nu_{1}^{\Lambda }$, so

(62) $$\Delta \nu \left(\Omega_{1},\nu_{1,*}^{\Lambda },\Lambda_{*},r\right)\approx -k_{1}\left(r\right)\Delta \nu \left(-k_{2}\left(r\right)\Omega_{1},\nu_{1,*}^{\Lambda },\Lambda_{*}\right)$$

with $k_{1}\left(r\right),k_{2}\left(r\right)\in R\setminus \left\{0\right\}$ is a possible model to interpret the data in Figure 3c.

As shown in Figure 3b,d,f, the behavior of Δν with respect to the latitude of the emitter Λ was always decreasing, so

(63) $$\frac{\partial \left(\Delta \nu \right)}{\partial \Lambda }<0$$

in every case.

In Figure 3e, the behavior of Δν with respect to the combined variation of both the orbit radius *r* and the true anomaly of the first satellite scaled with the actual position of the emitter $\nu_{1}^{\Lambda }$ resembles a similar amplification and a change of scale with respect to *r* of (62), but without anti-symmetry, as shown in (64), where $k_{1}\left(r\right),k_{2}\left(r\right)\in R\setminus \left\{0\right\}$.

(64) $$\Delta \nu \left(\Omega_{1,*},\nu_{1}^{\Lambda },\Lambda_{*},r\right)\approx k_{1}\left(r\right)\Delta \nu \left(\Omega_{1,*},k_{2}\left(r\right)\nu_{1}^{\Lambda },\Lambda_{*}\right)$$

Figure 3f shows the decreasing behavior of Δν with respect to Λ, as stated in (63), but also reveals a minimal (if not negligible) effect of the orbit radius *r* when $\Omega_{1}$ and $\nu_{1}^{\Lambda }$ were not varying, so $\Delta \nu \left(\Omega_{1,*},\nu_{1,*}^{\Lambda },\Lambda ,r\right)\approx \Delta \nu \left(\Omega_{1,*},\nu_{1,*}^{\Lambda },\Lambda \right)$.

The third set of functions were the ones reported in (54). From Figure 4a–c, the dependence of $\gamma_{1,2}$ with respect to $\Omega_{1}$ reveals macroscopic symmetric behavior, but with nonlinear influences due to other variables.

The completely symmetric model predicted in [26], i.e., $\gamma_{1,2}\left(\Omega_{1}\right)=\gamma_{1,2}\left(-\Omega_{1}\right)$, obtained in simplified scenarios, needed to be corrected with nonlinear influences that depended on the actual values of $\nu_{1}^{\Lambda }$, Λ, and *r*.

Possible additive models could be the ones reported in (65)–(67).

(65) $$\gamma_{1,2}\left(\Omega_{1},\nu_{1}^{\Lambda },\Lambda_{*},r_{*}\right)=\gamma_{1,2}\left(-\Omega_{1},\nu_{1}^{\Lambda },\Lambda_{*},r_{*}\right)+f\left(\nu_{1}^{\Lambda },\Lambda_{*},r_{*}\right)$$

(66) $$\gamma_{1,2}\left(\Omega_{1},\nu_{1,*}^{\Lambda },\Lambda ,r_{*}\right)=\gamma_{1,2}\left(-\Omega_{1},\nu_{1,*}^{\Lambda },\Lambda ,r_{*}\right)+f\left(\nu_{1,*}^{\Lambda },\Lambda ,r_{*}\right)$$

(67) $$\gamma_{1,2}\left(\Omega_{1},\nu_{1,*}^{\Lambda },\Lambda_{*},r\right)=\gamma_{1,2}\left(-\Omega_{1},\nu_{1,*}^{\Lambda },\Lambda_{*},r\right)+f\left(\nu_{1,*}^{\Lambda },\Lambda_{*},r\right)$$

From Figure 4a,d, the behavior of $\gamma_{1,2}$ with respect to the true anomaly of the first satellite $\nu_{1}^{\Lambda }$ (translated considering the actual latitude of the emitter) revealed two distinct trends: $\gamma_{1,2}$ was decreasing for negative values of $\nu_{1}^{\Lambda }$ and $\gamma_{1,2}$ was increasing for positive values of $\nu_{1}^{\Lambda }$, so

(68) $$\begin{array}{cc} \frac{\partial \left(\gamma_{1,2}\right)}{\partial \nu_{1}^{\Lambda }}>0 & \nu_{1}^{\Lambda }>0\\ \frac{\partial \left(\gamma_{1,2}\right)}{\partial \nu_{1}^{\Lambda }}<0 & \nu_{1}^{\Lambda }<0 \end{array}$$

is a possible interpretation of the data.

However, the trends in Figure 4a,d are related to low altitudes (i.e., h=400 km); when the value of the altitude became higher, the main trend was the increasing one, as shown in Figure 4e, revealing a possible multiplicative influence of the orbit radius *r*. Given two orbit radii $r_{S_{A}}$ and $r_{S_{B}}$, a possible model is the one shown in (69), where $\Delta r_{A,B}=r_{S_{B}}-r_{S}A$ is the difference of the orbit’s radii, *f* is multiplicative function of the orbit radii, and k∈R is an eventual additive shift. In particular, f<0 for $\nu_{1}^{\Lambda }<0$ and f>0 for $\nu_{1}^{\Lambda }>0$. 5

(69) $$\gamma_{1,2}\left(r_{S_{B}}\right)\approx f\left(\nu_{1}^{\Lambda },\Delta r_{A,B}\right)\gamma_{1,2}\left(r_{S_{A}}\right)+k\left(\nu_{1}^{\Lambda },\Delta r_{A,B}\right)$$

From Figure 4b, the behavior of $\gamma_{1,2}$ with respect to Λ, varying $\Omega_{1}$ but keeping a fixed value of $\nu_{1}^{\Lambda }$ and orbit radius *r*, revealed an increase in the spacing angle, i.e., given $\Lambda_{A}>\Lambda_{B}$, then

(70) $$\gamma_{1,2}\left(\Omega_{1},\nu_{1,*}^{\Lambda },\Lambda_{A},r_{*}\right)>\gamma_{1,2}\left(\Omega_{1},\nu_{1,*}^{\Lambda },\Lambda_{B},r_{*}\right)$$

for every value of $\Omega_{1}$.

From Figure 4d, the behavior of $\gamma_{1,2}$ with respect to Λ, varying $\nu_{1}^{\Lambda }$ but keeping a fixed value of $\Omega_{1}$ and orbit radius *r*, revealed two trends: $\gamma_{1,2}$ was increasing for Λ>0 and decreasing for Λ<0, so (71) can be formulated as a macroscopic level of approximation.

(71) $$\begin{array}{cc} \frac{\partial \left(\gamma_{1,2}\right)}{\partial \nu_{1}^{\Lambda }}\geq 0 & \Lambda >0\\ \frac{\partial \left(\gamma_{1,2}\right)}{\partial \nu_{1}^{\Lambda }}\geq 0 & \Lambda <0 \end{array}$$

Considering also the specular trends shown in Figure 4d, the property in (72) holds.

(72) $$\gamma_{1,2}\left(\Omega_{1,*},\nu_{1}^{\Lambda },\Lambda ,r_{*}\right)=-\gamma_{1,2}\left(\Omega_{1,*},\nu_{1}^{\Lambda },-\Lambda ,r_{*}\right)$$

Regarding the final result of this study, Figure 4f shows the combined influence of the latitude of the emitter Λ and the orbit radius *r* when the angular position of the first satellite was fixed and not varying (i.e., $\Omega_{1}=\Omega_{1,*}$ and $\nu_{1}^{\Lambda }=\nu_{1,*}^{\Lambda }$). In particular, the effect of Λ was dominant with a second order trend and a local minimum; instead, the effect of the orbit radius *r* appeared to be decoupled. Properties in (73) held with $\Lambda_{m}\left(\Omega_{1,*},\nu_{1,*}^{\Lambda }\right)$, the value of the minimum that depended on the actual values of $\Omega_{1}$ and $\nu_{1}^{\Lambda }$.

(73) $$\begin{array}{cc} \frac{\partial \left(\gamma_{1,2}\right)}{\partial \Lambda }>0 & \Lambda >\Lambda_{m}\left(\Omega_{1,*},\nu_{1,*}^{\Lambda }\right)\\ \frac{\partial \left(\gamma_{1,2}\right)}{\partial \Lambda }<0 & \Lambda <\Lambda_{m}\left(\Omega_{1,*},\nu_{1,*}^{\Lambda }\right)\\ \frac{\partial^{2}\left(\gamma_{1,2}\right)}{\partial \Lambda^{2}}<0 & \forall \Lambda \in \left(-\frac{\pi }{2},\frac{\pi }{2}\right)\\ \frac{\partial \left(\gamma_{1,2}\right)}{\partial r}>0 & \forall \Lambda \in \left(-\frac{\pi }{2},\frac{\pi }{2}\right) \end{array}$$

## 4. Conclusions

This paper provided an in-depth study on the optimal distribution of satellite constellations and offers a theoretical framework applicable to clusters of arbitrary numbers of satellites. For Earth scanning missions with circular orbits and target pointing attitude, this study developed a mathematical model to optimize the relative distribution of an arbitrary number of satellites equipped with receivers capable of performing AOA measurements.

Extensive simulations were performed for systems composed of two satellites with the purpose of geolocating one signal emitter located on the surface of Earth. In particular, quantitative results were presented in terms of differences in the angles of the right ascension of the ascending node ΔΩ, differences in the angles of the true anomaly Δν, and the spacing angle $\gamma_{1,2}$ between the satellites with respect to the emitter position. Qualitative trends were also discussed, which fully confirm the previously predicted properties of symmetry and anti-symmetry for the difference in the right ascension of the ascending node ΔΩ and the difference in true anomalies Δν and partially confirm symmetric properties for spacing angle γ obtained in a previous study by the authors [26] while taking into account variation of the configurations in terms of orbit radius *r* and the position of the emitter in terms of latitude Λ.

This research provides a guide for translating mission planning requirements into satellite constellation designs for the operative case of the placement of a second companion satellite after a first one is launched.

The theoretical analysis of the trends of two-satellite systems presented in this paper can also support the building of data-driven fitting and modeling for simulators, facilitating fast predictions of constellation designs, at least for two satellites, and configuration comparisons, providing beneficial insights for decision-makers in the space domain.

It is noted that among the limitations of this study to be addressed in future activities are the influence of the attitude of the satellites and the effect due to the signal-to-noise ratio. Also, third party validation of the results and comparison with other optimization approaches or strategies to model the constraints could be explored in future works. Furthermore, future studies could expand on this work by simulating constellations with more satellites, incorporating multiple measurements over time, and exploring more complex scenarios by relaxing some of the assumptions made in this study.

## Figures and Tables

**Table 1:** Analysis of the precision of the results with different settings of the solver.

Settings $\left(T_{s},T_{o},T_{c}\right)$	Solution [°]	Precision
$\left(10^{-3},10^{-3},10^{-3}\right)$	$\Delta \Omega_{1}=7.79988394977692678367021$	$10^{-5}$
	$\Delta \nu_{1}=-22.2001160502229595294921$	$10^{-6}$
	$\gamma_{1,2}=46.9567469983894341112318$	$10^{-5}$
$\left(10^{-6},10^{-6},10^{-6}\right)$	$\Delta \Omega_{1}=7.79989639975224235968199$	$10^{-7}$
	$\Delta \nu_{1}=-22.2001036002477576403180$	$10^{-8}$
	$\gamma_{1,2}=46.9567650909294656003112$	$10^{-8}$
$\left(10^{-9},10^{-9},10^{-9}\right)$	$\Delta \Omega_{1}=7.79989630705961189960362$	$10^{-10}$
	$\Delta \nu_{1}=-22.2001036929402744135587$	$10^{-11}$
	$\gamma_{1,2}=46.9567649562266637985886$	$10^{-10}$
$\left(10^{-12},10^{-12},10^{-12}\right)$	$\Delta \Omega_{1}=7.79989630740783468354493$	<$10^{-40}$
	$\Delta \nu_{1}=-22.2001036925921653164551$	<$10^{-40}$
	$\gamma_{1,2}=46.9567649567326341752960$	<$10^{-40}$
$\left(10^{-15},10^{-12},10^{-12}\right)$	$\Delta \Omega_{1}=7.79989630740783468354493$	Reference
	$\Delta \nu_{1}=-22.2001036925921653164551$	Reference
	$\gamma_{1,2}=46.9567649567326341752960$	Reference

## Data Availability

Dataset available upon request.

## Abbreviations

The following abbreviations are used in this manuscript:

AOA	Angle of Arrival
CRLB	Cramér–Rao Lower Bound
DOP	Dilution of Precision
ECEF	Earth-Centered Earth-Fixed Coordinate System
ECI	Earth-Centered Inertial Coordinate System
GNSS	Global Navigation Satellite System
KKT	Karush–Kuhn–Tucker Conditions
LEO	Low Earth Orbit
LOB	Line of Bearing
LOS	Line of Sight
LLH	Latitude, Longitude, Height Coordinate System
PDOP	Position Dilution of Precision
PQW	Perifocal Coordinate System

## Appendix A. Mathematical Notation

*a*	Semi-major axis of the elliptic orbit of the satellite
$a_{⊕}$	Elipsoidal equatorial radius
*c*	Speed of light
C	Covariance Matrix
D	Domain of the objective function
*e*	Eccentricity of the elliptic orbit of the satellite
$e_{⊕}$	Eccentricity of the Earth’s ellipsoid
*E*	Emitter point
*f*	Frequency of the signal
F	Matrix of Fisher
*g*	Nonlinear objective function
*h*	Height
H	Geometric Design Matrix
*i*	Inclination of the orbit of the satellite
I	Identity matrix
*L*	Total length of linear array of antennas

*m*	Number of nonlinear inequality constraint conditions
*M*	Number of elements of linear array of antennas
*n*	Total number of satellites
*N*	Auxiliary function to describe ellipsoidal Earth model
{p,q,w}	Axes of the perifocal coordinate system
PDOP	Position Dilution of Precision
*r*	Radius of circular orbit of the satellite
$r_{*}$	Radius of circular orbit of the satellite (fixed value)
$r_{⊕}$	Radius of Earth (spherical model)
R	Matrix of rotation
*s*	Auxiliary variable (used in Geometric Design Matrix)
*S*	Satellite point
SNR	Signal-to-noise ratio
*t*	Time
$T_{c}$	Constraint tolerance
$T_{o}$	Optimality tolerance
$T_{s}$	Step tolerance
*u*	Auxiliary variable (used in Geometric Design Matrix)
$v_{\mathrm{LOB}}$	Unit vector of line of bearing
*w*	Nonlinear inequality constraint function
{x,y,z}	Set of cartesian coordinates
x	Position vector
$\alpha_{\mathrm{Az}}$	Azimuth angle
$\alpha_{\mathrm{El}}$	Elevation angle
$\alpha_{\mathrm{GR}}$	Angular position of Greenwich meridian
γ	Spacing angle
δ	Maximum angle of line of sight
$\delta_{r}$	Maximum angle of line of sight (relative)
Δ	Difference operation
$\varepsilon_{\mathrm{Az}}$	Complementary of Azimuth angle
$\varepsilon_{\mathrm{El}}$	Complementary of Elevation angle
λ	Longitude
Λ	Geodetic latitude
$\Lambda_{*}$	Geodetic latitude (fixed value)
ν	True anomaly of the orbit
$\nu^{\Lambda }$	True anomaly of the orbit (relative value to Nadir alignment)
$\nu_{0}$	True anomaly of the orbit (Nadir alignment)
$\nu_{*}$	True anomaly of the orbit (fixed value)
ω	Argument of perigee of the orbit of the satellite
Ω	Right ascension of the ascending node
$\Omega_{0}$	Right ascension of the ascending node (Nadir alignment)
$\Omega_{*}$	Right ascension of the ascending node (fixed value)
σ	Standard deviation
